# Identifying data anomalies in milk component measurements from partial-day milking records

**DOI:** 10.3168/jdsc.2025-0825

**Published:** 2025-12-13

**Authors:** Xiao-Lin Wu, Malia J. Caputo, Chip Donatone, Asha M. Miles, Ransom L. Baldwin, Steven Sievert, Jay Mattison, John B. Cole, Javier Burchard, João Dürr

**Affiliations:** 1 Council on Dairy Cattle Breeding, Bowie, MD 20716; 2 Department of Animal and Dairy Sciences, University of Wisconsin–Madison, Madison, WI 53706; 3 USDA Animal Genomics and Improvement Laboratory, Beltsville, MD 29705; 4 National Dairy Herd Information Association, Verona, WI 53711; 5 Department of Animal Science, North Carolina State University, Raleigh, NC 27607; 6 Department of Animal Sciences, Donald Henry Barron Reproductive and Perinatal Biology Research Program, and the Genetics Institute, University of Florida, Gainesville, FL 32611; 7 Department of Animal Biosciences, University of Guelph, Ontario, Canada N1G 2W1

## Abstract

High-quality milk and milk component data are crucial for accurate genetic evaluations and effective herd management. However, data recording errors can compromise the validity of downstream decisions. In a recent study, we proposed using intraclass correlation coefficients as a herd-level metric to assess the consistency of milk components from single milkings, thereby effectively identifying farms with potential data quality concerns. A key challenge, however, is whether potentially erroneous records can be detected at the cow-day level. In this study, we introduce a novel metric—individual-level intraclass correlations—to assess data consistency at the cow-day level and evaluate its performance against 3 commonly used anomaly-detection methods. We further introduce a 2-step approach to estimate percentile thresholds for flagging outliers. The results demonstrate the superior performance of this new metric over the conventional univariate and multivariate methods in identifying anomalies in correlated partial daily milk component data. In addition, the negative impact of data shuffling was examined. Together, these methods provide robust and practical tools for detecting suspect milk component records at the individual cow-day level.

## Highlights

• Record shuffling, like other milking errors, reduces accuracy of daily milking records.
• Conventional uni- and multivariate methods struggle to detect issues in correlated milk data.
• The new metric is effective for flagging anomalies in cow-day milk component records.
• We introduce a 2-step approach to estimate percentile thresholds as cutoffs.

## Graphical Abstract

**Figure:**
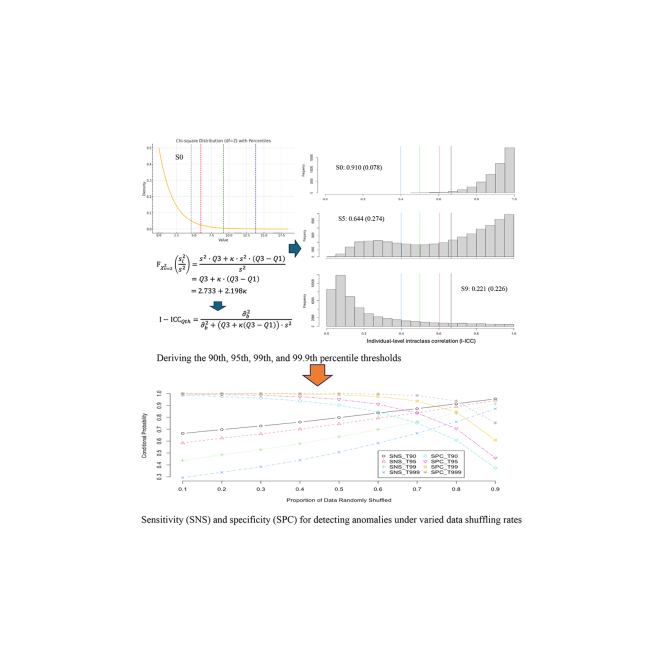
**Summary:** High-quality milk and milk component data are essential for accurate genetic evaluations and effective daily herd management. In a recent study, we demonstrated the usefulness of intraclass correlation coefficients as a herd-level metric for assessing the consistency of fat and protein percentages from single milkings. However, a key challenge remains: How can we detect potentially erroneous records at the individual cow-day level? In this study, we introduced a new metric—individual-level intraclass correlations—to assess data quality at the cow-day level. We evaluated its performance in comparison to 3 commonly used methods.

High-quality milk and milk component data are crucial for accurate genetic evaluations and effective routine herd management. However, potential errors can compromise the reliability of these data, leading to inconsistent conclusions and even incorrect decisions. In a recent study, we demonstrated the use of intraclass correlation coefficients (**ICC**) as a herd-level metric to assess the consistency of milk component data quality (Wu et al., 2025a). Statistically, ICC extends the concept of pairwise correlation to situations involving more than 2 observations per group and quantifies the degree of similarity among them (Harris, 1913; Fisher, 1954). It has been widely used to evaluate measurement reliability and inter-rater consistency across grouped data (Bartko, 1966). In human and animal genetics, ICC can serve as a metric for assessing repeatability (Kumro et al., 2021) and heritability (Visscher, 1992). In the latter applications, for example, the intraclass correlation approximately equals 1/2 heritability under balanced nested full-sib designs, or 1/4 heritability under balanced nested designs (Falconer and Mackay, 1996).

Outlier detection is a common technique in statistical analysis that aims to identify data anomalies. An outlier refers to a data point that deviates markedly from other observations and may result from natural data variability, novel or unexpected events, or measurement errors (Grubbs, 1969). The process of detecting outliers depends on the nature and distribution of the data, as well as the specific context in which the data are generated (Barnett and Lewis, 1994). A univariate outlier is an extreme value in the context of a single variable. For example, a cow produces an extraordinarily high milk yield compared with the population average. In contrast, multivariate outliers are observations that appear unusual only when considering multiple variables simultaneously. For example, a cow may produce a normal fat percentage (**FP**) in one milking but an abnormally low or high FP in another on the same test day, although both numbers may fall within the ranges of their respective milkings.

In this paper, we introduce a novel metric for identifying data anomalies at the cow-day level and evaluate its performance against 3 commonly used methods (Grubbs, 1969).

Let *x_ij_* be an observed value for individual *i* (where *i* = 1, . . ., *n*) in milking *j* (where *j* = 1, . . ., *m*). The z-score approach (Grubbs, 1969) computes the standardized deviation for each observed value as follows:

[1] $$z_{ij}=\frac{x_{ij}-{\bar{x}}_{i}}{\sqrt{{\hat{\sigma }}_{within}^{2}}}.$$

The estimated within-group variance ${\hat{\sigma }}_{within}^{2}$ is calculated as follows:

[2] $${\hat{\sigma }}_{within}^{2}=\frac{1}{n\left(m-1\right)}\sum_{i=1}^{n}\sum_{j=1}^{m}{\left(x_{ij}-{\bar{x}}_{i}\right)}^{2},$$

where ${\bar{x}}_{i}=\frac{1}{m}\sum_{j=1}^{m}x_{ij}.$

Statistically, the z-score approach assumes a standard normal distribution. Thus, using $\left(z\right)>3$ corresponds to the 99.73rd percentile, meaning that ∼0.27% of the observations may fall outside this threshold. Alternatively, using $\left(z\right)>2$ (or $\left(z\right)>4)$ corresponds to the 95.45th (or 99.99th) percentile, flagging ∼4.55% (or 0.0063%) as outliers.

The interquartile range (**IQR**) approach (Tukey, 1977) is a nonparametric method that works even when the data do not follow a normal distribution. The IQR is calculated as

[3] IQR = Q3 − Q1,

where Q1 is the 25th percentile and Q3 is the 75th percentile of *x_ij_*.

Outlier thresholds are often defined using a multiplier of *κ* = 1.5. That is,

[4] *d_ij_* < Q1 − *κ*·IQR or *d_ij_* > Q3 − *κ*·IQR.

For a standard normal distribution, applying an IQR multiplier of 1.5 corresponds to the 99.65th percentile, implying that about 0.35% of values in the upper tail may fall beyond this threshold. Alternative multipliers, such as *κ* = 1.0, 2.0, or 3.0, can be used depending on whether more conservative or aggressive detection is desired.

The Mahalanobis distance (**MD**) is a multivariate measure of the distance from a point to the multivariate mean, taking into account correlations among variables. Unlike Euclidean distance, which treats each dimension independently, the MD accounts for the covariance structure of the data, making it more suitable for detecting multivariate outliers. Let **x** be a *k*-dimensional observation vector (e.g., corresponding to *k* milkings), **μ** be the corresponding mean vector, and **S** be the sample covariance matrix across the *k* milkings. The squared MD is given by

[5] $${\text{D}}_{\text{M}}^{2}\left(\text{x}\right)={\left(\text{x}-\mu \right)}^{“}{\text{S}}^{-1}\left(\text{x}-\mu \right).$$

Under a multivariate normal distribution assumption for the data, the squared Mahalanobis distances $D_{M}^{2}\left(x\right)$ ​follow a chi-squared distribution with *k* degrees of freedom:

[6] $$D_{M}^{2}\left(x\right)\&sim;\chi_{k}^{2}.$$

Hence, outliers can be identified by comparing them to a chi-squared distribution: $D_{M}^{2}\left(x\right)>\chi_{k,\;1-\alpha }^{2},$ where, for example, *α* = 0.05 or 0.01.

In this study, we introduce a new metric, namely the individual-level intraclass correlation coefficient (**I-ICC**), for identifying potential data anomalies in daily milk component measurements at the cow-day level. Consider the following one-way random effects model:

[7] *x_ij_* = *μ* + *b_i_* + *ε_ij_*,

where *μ* is the overall mean, $b_{i}\&sim;N\left(0,\sigma_{b}^{2}\right)$ is the random effect of group *i* with mean zero and variance $\sigma_{b}^{2},$ and ${}_{ij}\&sim;N\left(0,\sigma_{w}^{2}\right)$ denotes the within-group residual deviation from the group mean. Here, a group refers to a unique combination of a cow and a specific DIM.

The variance of an individual measurement *x_ij_* is given by

[8] $$Var\left(x_{ij}\right)=\sigma_{b}^{2}+\sigma_{w}^{2},$$

where the within- and between-group variances are estimated as follows:

[9] $${\hat{\sigma }}_{w}^{2}=\frac{1}{n\left(m-1\right)}\sum_{i=1}^{n}\sum_{j=1}^{m}{\left(x_{ij}-{\bar{x}}_{i}\right)}^{2},$$

[10] $${\hat{\sigma }}_{b}^{2}=\frac{1}{n-1}\sum_{i=1}^{n}{\left({\bar{x}}_{i}-\bar{x}\right)}^{2}-\frac{1}{m}{\hat{\sigma }}_{w}^{2},$$

where ${\bar{x}}_{i}$ and $\bar{x}$ stand for the mean of group *i* and the population mean, respectively.

The herd-level ICC is then computed as follows (Wu et al., 2025a):

[11] $$\mathrm{ICC}=\frac{{\hat{\sigma }}_{b}^{2}}{{\hat{\sigma }}_{b}^{2}+{\hat{\sigma }}_{w}^{2}},$$

which quantifies the consistency of repeated measurements of cows in a herd. A low ICC value reflects high within-group variability and inconsistency, thereby warranting further examination of the data.

Following similar logic, the I-ICC is defined by replacing the global estimate of within-group variance $\left({\hat{\sigma }}_{w}^{2}\right)$ in Equation 11 with its component corresponding to each cow-day group $\left(s_{i}^{2}\right).$ That is,

[12] $$I-\mathrm{IC}C_{i}=\frac{{\hat{\sigma }}_{b}^{2}}{{\hat{\sigma }}_{b}^{2}+s_{i}^{2}},$$

where $s_{i}^{2}=\frac{1}{m-1}\sum_{j=1}^{m}{\left(x_{ij}-{\bar{x}}_{i}\right)}^{2}$ is the observed within-group sample variance across *m* milkings for a specific cow-day.

Directly defining the threshold for flagging I-ICC outliers using Equation 12 is statistically challenging because it does not follow a standard distribution. Here, we propose a 2-step approach. The first step uses the theoretical chi-squared distribution to analytically derive the upper-boundary threshold (i.e., the 99.9th percentile) for individual within-group variance under the null hypothesis. Next, this variance threshold is then used to compute the corresponding percentile of the I-ICC distribution using Equation 12.

Let the within-group residuals follow a normal distribution and be independent across milkings: $e_{ij}\&sim;N\left(0,\sigma_{w}^{2}\right).$ Denote the standardized residuals as $z_{ij}=\frac{x_{ij}-{\bar{x}}_{i}}{\sigma_{w}}.$ Then, $\sum_{j=1}^{m}z_{ij}^{2}$ follows a chi-squared distribution with *m* − 1 degrees of freedom:

[13] $$\sum_{j=1}^{m}z_{ij}^{2}=\sum_{j=1}^{m}{\left(\frac{x_{ij}-{\bar{x}}_{i}}{\sigma_{w}}\right)}^{2}\&sim;\chi_{m-1}^{2}.$$

The within-group sample variance for group *i*, $s_{i}^{2}$ is then a scaled chi-squared distribution:

[14] $$s_{i}^{2}=\frac{1}{m-1}\sum_{j=1}^{m}{\left(x_{ij}-{\bar{x}}_{i}\right)}^{2}=\frac{\sigma_{w}^{2}}{m-1}\sum_{j=1}^{m}{\left(\frac{x_{ij}-{\bar{x}}_{i}}{\sigma_{w}}\right)}^{2}\&sim;\frac{\sigma_{w}^{2}}{m-1}\cdot \chi_{m-1}^{2},$$

where the scale parameter is *s*^2^ = σw2σw2m-1m-1, and *m* − 1 denotes the degrees of freedom.

To develop an outlier flagging criterion in the second step, we use an IQR-based approach. Specifically, we let $s_{\left(Q99.9\%\right)}^{2}$ denote the 99.9th percentile of $s_{i}^{2},$ and express this percentile as a linear function of the interquartile range,

[15] $$s_{\left(Q99.9\%\right)}^{2}=Q3+\kappa \cdot \left(Q3-Q1\right),$$

where *κ* is the IQR multiplier to be determined.

Substituting in terms of chi-squared quantiles gives

$$s_{\left(Q99.9\%\right)}^{2}=s^{2}\cdot \chi_{m-1\left(Q3\right)}^{2}+\kappa \cdot s^{2}\cdot \left(\chi_{m-1\left(Q3\right)}^{2}-\chi_{m-1\left(Q1\right)}^{2}\right).$$

For example, with *m* = 3 (i.e., 3 milkings daily per cow), we have $\chi_{2\left(Q3\right)}^{2}=2.773$ and $\chi_{2\left(Q1\right)}^{2}=0.575.$ Thus,

[16] $$s_{\left(Q99.9\%\right)}^{2}=\left(2.773+2.198\kappa \right)\cdot s^{2}.$$

To determine *κ*, we solve for the value that ensures the upper cutoff corresponds to the 99.9th percentile, which is defined by the following probability (Pr):

[17] $$\mathrm{Pr}\left(s_{i}^{2}\leq \left(2.773+2.198\kappa \right)\cdot s^{2}\right)=0.999.$$

Rearranging the left-hand side of the above inequality and noting that $\frac{s_{i}^{2}}{s^{2}}\&sim;\chi_{m-1}^{2},$ we have

[18] Fχm-122.773+2.198κ=:Prsi2si2s2≤2.773+2.198κs2≤2.773+2.198κ=0.999.

Here, $F_{\chi_{m-1}^{2}}\left(x\right)$ represent the cumulative distribution function (**CDF**) of the chi-squared distribution with *m* −1 degrees of freedom, evaluated at *x*.

Reverting the CDF gives 2.773 + 2.198*κ* = $F_{\chi_{2}^{2}}^{-1}\left(0.999\right)$ ≈ 13.82, and the 99.9th percentile threshold is obtained as *κ* = $\frac{13.82-2.773}{2.198}$ ≈ 5.023. Similarly, the one-sided 90th, 95th, and 99th percentile thresholds for detecting outliers in $s_{i}^{2}$ are 0.834, 1.464, and 2.93, respectively (see the Graphical Abstract for illustration). Importantly, these IQR multipliers are independent of the scaling parameter $s_{i}^{2}$ because it cancels out in Equation 18, thus providing convenience to practical implementation.

Computing the κ multiplier value is easy, as illustrated by the following R function:

kappa_IQR <- function(m, q) { df <- m - 1 Q1 <- qchisq(0.25, df) Q3 <- qchisq(0.75, df) Qq <- qchisq(q, df) return((Qq - Q3) / (Q3 - Q1))}

In the second step, the corresponding I-ICC threshold is computed as follows:

[19] $$I-\mathrm{IC}C_{i}<r_{Q99.9\%}=\frac{{\hat{\sigma }}_{b}^{2}}{{\hat{\sigma }}_{b}^{2}+s_{\left(Q99.9\%\right)}^{2}}=\frac{{\hat{\sigma }}_{b}^{2}}{{\hat{\sigma }}_{b}^{2}+\left(2.773+2.198\kappa \right)\cdot s^{2}}.$$

Any I-ICC value smaller than the threshold is considered an outlier.

We evaluated the performance of this new metric, along with the 3 commonly used methods. The milk component data were collected from 4 Holstein dairy farms (A, B, C, and D) practicing 3 times daily (**3×**) milking in 3 US states. In this study, milk yields and components from 3× milkings were measured weekly for each cow up to 120 d, followed by monthly tests until 305 d or the end of lactation. After cleaning the data to remove duplicates and missing or incomplete data, we retained 15,995 × 3 (A), 13,336 × 3 (B), 8,363 × 3 (C), and 11,182 × 3 (D) milking records for subsequent analyses.

To investigate the effects of data shuffling, we also simulated a pooled dataset (S0) containing 48,876 × 3 milking records without errors for each trait using multivariate normal distributions. The means and the variance-covariance matrices for the 3 milkings were set to be weighted averages across the 4 Holstein dairy farms. We also generated 9 pooled datasets (designated S1 to S9) for each trait by randomly shuffling between 10% (S1) and 90% (S9) of the records among cows per farm on each milking section.

In practice, record shuffling may occur due to improper labeling of the milk sample vial, misplacement of the milk sample vial in the sample rack, or collecting extra or missing milk samples after the cow ID has already been recorded at farms. Once samples arrive at the laboratory, additional errors may be introduced by adding samples out of order or misaligning the sample rack when moving milk samples from the sample rack to the instrument. Errors in merging the cow ID with the corresponding sample ID can further contribute to record shuffling. All methods showed low outlier rates in the 4 farms, ranging from 0.33% to 6.55% for FP (Table 1) and 0.44% to 5.02% for protein percentage (**PP**; Table 2). Based on the weighted variance components across the 4 herds, the percentile thresholds for I-ICC were computed as: 0.087 (99.9th), 0.125 (99th), 0.180 (95th), and 0.223 (90th) for FP; and 0.387 (99.9th), 0.486 (99th), 0.593 (95th), and 0.654 (90th) for PP. All 4 Holstein herds had high I-ICC values on average. In these 4 farms, the ICC ranged from 0.508 to 0.666 for FP and 0.875 to 0.927 for PP, whereas the I-ICC ranged from 0.681 to 0.801 for FP and 0.926 to 0.943 for PP (Table 1, Table 2). The higher ICC and I-ICC values for PP suggest that protein percentage records are inherently more consistent among cows than FP records.

**Table 1:** Summary statistics and outlier rates (%) of milk fat percentage using 4 methods in 4 Holstein herds (A, B, C, and D) and 10 simulated datasets (S0-S9)^1^

Dataset	n	Mean (%)	${\hat{\sigma }}_{b}^{2}$	${\hat{\sigma }}_{w}^{2}$	ICC	I-ICC	Outliers^2^ (%)						
							z-score	IQR	MD	I-ICC90	I-ICC95	I-ICC99	I-ICC999
A	15,995	4.04	0.274	0.244	0.529	0.681 (0.233)	0.75	1.14	4.55	4.55	2.96	1.37	0.54
B	13,336	4.05	0.308	0.154	0.666	0.801 (0.208)	0.48	0.73	2.81	2.41	1.49	0.73	0.29
C	8,363	4.51	0.256	0.248	0.508	0.699 (0.239)	0.97	1.40	4.63	5.03	3.50	1.94	1.05
D	11,182	4.15	0.368	0.283	0.565	0.717 (0.236)	1.09	1.69	6.55	4.24	2.55	1.03	0.33
S0	48,876	4.15	0.308	0.211	0.593	0.667 (0.200)	0.01	0.06	1.12	0.60	0.13	<0.01	<0.01
S1	48,876	4.15	0.278	0.242	0.534	0.630 (0.215)	0.16	0.33	2.60	2.45	1.10	0.24	0.04
S2	48,876	4.15	0.246	0.274	0.473	0.590 (0.228)	0.32	0.63	4.15	5.23	2.77	0.78	0.15
S3	48,876	4.15	0.215	0.305	0.413	0.546 (0.238)	0.47	0.92	5.70	9.19	5.36	1.78	0.41
S4	48,876	4.15	0.184	0.336	0.354	0.500 (0.245)	0.61	1.19	7.17	14.5	9.16	3.53	0.98
S5	48,876	4.15	0.153	0.367	0.294	0.448 (0.248)	0.76	1.47	8.71	22.0	15.1	6.77	2.35
S6	48,876	4.15	0.121	0.399	0.233	0.389 (0.246)	0.92	1.76	10.2	32.0	23.9	12.6	5.39
S7	48,876	4.15	0.091	0.429	0.174	0.323 (0.237)	1.05	2.04	11.7	44.9	36.1	22.3	11.7
S8	48,876	4.15	0.059	0.461	0.113	0.243 (0.217)	1.21	2.34	13.3	61.6	53.6	39.2	25.6
S9	48,876	4.15	0.029	0.491	0.055	0.148 (0.175)	1.37	2.62	14.8	80.6	75.6	65.4	53.3

1 n = number of observation trios; ${\hat{\sigma }}_{w}^{2}$ = estimated within-group variance; ${\hat{\sigma }}_{b}^{2}$ = estimated between-group variance; ICC = herd-level intraclass correlation coefficients; I-ICC (SD) = mean (SD) of individual-level ICC. S0 = simulated dataset without data shuffling; S1–S9 = simulated datasets with data shuffling from 10% to 90%. Results for S0–S9 are presented as averages across 10 replicates.

2 z-score, IQR, MD, and I-ICCx = outlier rates detected using the z-score, interquartile range (IQR), Mahalanobis distance (MD), and the I-ICCx approach, where the threshold (x) is set to be the 90th, 95th, 99th, and 99.9th percentile, respectively.

**Table 2:** Summary statistics and outlier rates (%) of milk protein percentage using 4 methods in 4 real dairy farms (A, B, C, and D) and 10 simulated datasets (S0-S9)^1^

Dataset	n	Mean (%)	${\hat{\sigma }}_{b}^{2}$	${\hat{\sigma }}_{w}^{2}$	ICC	I-ICC	Outliers^2^ (%)						
							z-score	IQR	MD	I-ICC90	I-ICC95	I-ICC99	I-ICC999
A	15,995	3.09	0.087	0.007	0.927	0.943 (0.085)	0.44	0.60	1.69	1.83	1.33	0.79	0.50
B	13,336	3.19	0.088	0.010	0.897	0.929 (0.110)	1.03	1.32	3.27	3.65	2.88	1.80	1.14
C	8,363	3.25	0.114	0.012	0.903	0.940 (0.118)	1.73	2.11	5.02	4.27	3.42	2.33	1.34
D	11,182	3.18	0.103	0.015	0.875	0.926 (0.118)	1.39	1.78	4.53	4.25	3.13	1.91	1.14
C0	48,876	3.17	0.095	0.010	0.902	0.910 (0.077)	0.03	0.10	0.97	1.01	0.28	0.01	<0.01
C1	48,876	3.17	0.086	0.020	0.813	0.867 (0.149)	2.49	3.06	7.26	8.05	6.27	4.40	2.93
C2	48,876	3.17	0.076	0.029	0.721	0.820 (0.195)	5.00	6.08	13.6	15.9	13.2	9.76	6.76
C3	48,876	3.17	0.067	0.039	0.633	0.768 (0.229)	7.44	9.03	19.9	24.6	20.9	16.0	11.5
C4	48,876	3.17	0.057	0.048	0.543	0.710 (0.255)	9.93	12.0	26.2	34.1	29.6	23.4	17.6
C5	48,876	3.17	0.048	0.058	0.451	0.642 (0.276)	12.4	15.0	32.5	44.8	39.7	32.3	25.4
C6	48,876	3.17	0.038	0.067	0.359	0.565 (0.288)	15.0	18.1	38.8	56.4	51.2	42.9	35.2
C7	48,876	3.17	0.028	0.077	0.270	0.476 (0.290)	17.4	21.0	45.1	68.5	63.6	55.3	47.1
C8	48,876	3.17	0.019	0.086	0.180	0.368 (0.275)	19.9	24.0	51.4	80.9	77.0	69.9	62.2
C9	48,876	3.17	0.010	0.096	0.090	0.229 (0.229)	22.3	27.0	57.7	92.2	90.2	86.0	81.0

1 n = number of observation trios; ${\hat{\sigma }}_{w}^{2}$ = estimated within-group variance; ${\hat{\sigma }}_{b}^{2}$ = estimated between-group variance; ICC = herd-level intraclass correlation coefficients; I-ICC (SD) = mean (SD) of individual-level ICC. S0 = simulated dataset without data shuffling; S1–S9 = simulated datasets with data shuffling from 10% to 90%. Results for S0–S9 are presented as averages across 10 replicates.

2 z-score, IQR, MD, and I-ICCx = outlier rates detected using the z-score, interquartile range (IQR), Mahalanobis distance (MD), and the I-ICCx approach, where the threshold (x) is set to be the 90th, 95th, 99th, and 99.9th percentile, respectively.

For the synthetic dataset without recording errors (S0), the outlier rates were low (∼0%–1.12% for FP and 0%–1.01% for PP). The distribution of I-ICC showed a peak on the high-value end of the distribution (see S0 in the upper panel of the Graphical Abstract). Introducing mismatched data errors by shuffling records among cows progressively distorted the data structure, shifting the peak of I-ICC toward the low-value end of the distribution (see S5 and S9 in the upper panel of the Graphical Abstract). As the proportion of shuffled records increased from 10% to 90%, between-cow variance decreased and within-cow variance increased. As a result, the ICC dropped from 0.593 to 0.055 for FP and from 0.902 to 0.090 for PP. Similarly, the I-ICC declined from 0.667 to 0.148 for FP and from 0.910 to 0.229 for PP.

In the presence of data shuffling, the 2 univariate approaches tended to underestimate the outlier rates: 0.01% to 1.37% (FP) and 0.03% to 22.3% (PP) using the z-score method, and 0.06% to 2.62% (FP) and 0.10% to 27.0% (PP) using the IQR method. Univariate outlier detection methods are fundamentally limited to identifying extreme data values based solely on the variation of a single variable in isolation. However, milk component data are moderately to highly correlated across multiple milkings daily within cows. As a result, measurement errors or data mishandling may not appear abnormal when each milking is evaluated independently.

The MD test identified higher outlier rates: 1.12% to 14.79% for FP and 0.97% to 57.7% for PP, compared with the 2 univariate methods. The MD method leverages information from the variance-covariance matrix, capturing the relationships across the 3 milkings. Nevertheless, it still underestimated the true outlier proportions, given the magnitude of up to 90% of actual data shuffling in the synthetic datasets.

The estimated outlier rates obtained using the I-ICC approach most closely align with the simulated values among all methods (Table 1, Table 2). The percentile thresholds of I-ICC were computed based on the variance components from the simulated data without data shuffling: 0.096 (99.9th), 0.137 (99th), 0.196 (95th), and 0.241 (90th) for FP and 0.407 (99.9th), 0.508 (99th), 0.613 (95th), and 0.673 (90th) for PP. The identified outlier rate increased with the data shuffling rate, ranging from 0.04% to 2.45% under 10% data shuffling to 53.3% to 80.6% under 90% data shuffling for FP and from 2.93% to 8.05% with 10% data shuffling to 81.0% to 92.2% with 90% data shuffling for FP.

It is important to note that the identified outliers may not all be true outliers, and those not identified as outliers may not actually be non-outliers. By the nature of record shuffling, a small portion of them could retain accidental within-cow-day consistency. On the other hand, randomly shuffling records leads to decreased between-cow variance and underestimated I-ICC estimates. Hence, a portion of records with true I-ICC above the cutoff threshold can fall below the cutoff and, therefore, be falsely identified as anomalies.

We showed the estimated sensitivity and specificity rates for PP in the lower panel of the Graphical Abstract. Statistically, sensitivity is the conditional probability of observing a positive case when the actual status is positive (i.e., a true outlier), and specificity is the conditional probability of observing a negative case when the actual status is negative (Wu et al., 2025b). The probability of a false negative equals one minus the sensitivity, and the probability of a false positive equals one minus the specificity.

As the rate of record shuffling increased from 0.1 to 0.9, sensitivity increased from lower values (0.293–0.664) to 0.955 (90th percentile cutoff) and 0.872 (99.9th percentile cutoff), indicating a false negative rate between 0.045 and 0.128. Hence, the method became increasingly effective at detecting true anomalies as more records were mismatched, although a small fraction still went undetected. Meanwhile, specificity decreased from around 1.00 to 0.375 (90th percentile cutoff) and 0.753 (99.9th percentile cutoff), resulting in a false positive rate ranging from 0.625 to 0.247. This indicates that, with more extensive record shuffling, the method became more prone to incorrectly flagging normal records as outliers, possibly due to drastically reduced between-group variance. The changes in sensitivity and specificity for FP showed similar patterns; however, the false positive and false negative rates tend to be higher in general.

Using a lower percentile cutoff makes the method more aggressive, improving its ability to capture true outliers (higher sensitivity) but at the cost of elevated false positives or lower specificity. Conversely, a higher cutoff makes the method more conservative, ensuring that very few false positives occur, but at the risk of missing true outliers when the data are more severely corrupted. Therefore, selecting an appropriate percentile threshold should strike a balance between the tolerance for false alarms and the risk of overlooking actual data errors, taking into account the specific quality control goals.

Record shuffling, like many other milking record errors, compromised the accuracy of estimated daily FP and PP when estimated from partial daily records (Figure 1). We estimated daily fat (protein) percentage from each of the 3 milkings using a linear regression approach, which also included partial daily yield, partial daily fat (protein) percentage, milking interval time, DIM, and a categorical lactation effect as predictors. The R^2^ accuracy was computed as the true phenotypic variance over the sum of the true phenotypic variance and mean squared errors (**MSE**) evaluated by 3-fold cross-validation, and averaged across 3 milkings and 30 replicates. The R^2^ accuracy decreased from 0.806 to 0.385 for FP and from 0.941 to 0.349 as the record shuffling rate increased from 0% to 100% (Figure 1); the decrease rate was more drastic for PP than for FP. The MSE exceeded the true phenotypic variance, with a record shuffling rate larger than 60%.

**Figure 1:**
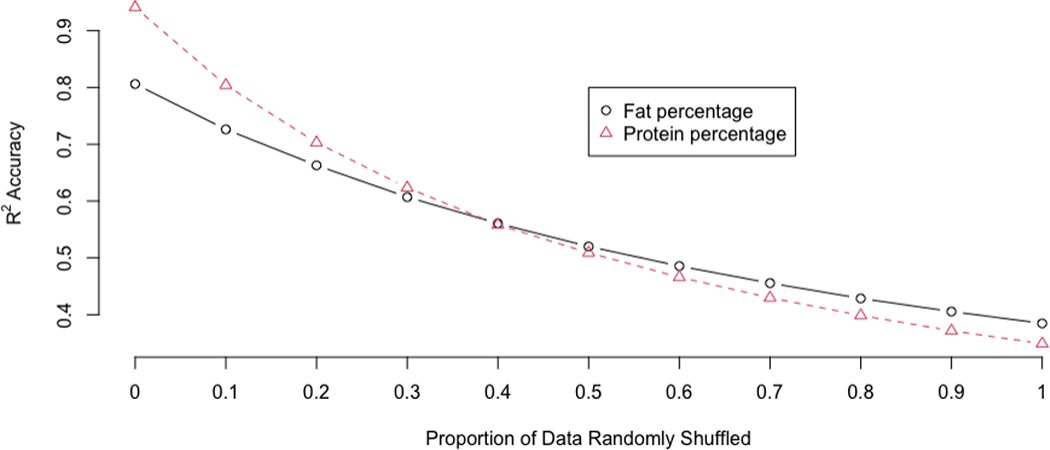
R^2^ accuracy of estimated daily fat and protein percentages using linear regression under varying record shuffling rates. $R^{2}=\frac{Var\left(y\right)}{Var\left(y\right)+MSE},$ where *Var*(*y*) = true phenotypic variance without measurement errors. MSE = mean squared error.

Finally, 2 issues are worth mentioning. First, because each cow-day group contains only 3 milkings (*m* = 3), the observed within-group variance $s_{i}^{2}$ is estimated from only 2 degrees of freedom and is subject to considerable sampling error. Alternatively, a simple empirical Bayes method can be used, which weighs $s_{i}^{2}$ and the global within-day variance component ${\hat{\sigma }}_{w}^{2}:$

[20] $${\tilde{s}}_{i}^{2}=w_{i}s_{i}^{2}+\left(1-w_{i}\right){\hat{\sigma }}_{w}^{2},w_{i}=\frac{m-1}{m-1+\tau },$$

where *τ* is a tuning constant chosen to balance individual and pooled information (typically *τ* ≈ 5 works well for *m* = 3). The resulting stabilized estimate of individual repeatability takes the same form as Equation 12, with $s_{i}^{2}$ replaced by ${\tilde{s}}_{i}^{2}.$

This empirical Bayes method provided more stable estimates of individual within-group variance, thereby yielding slightly smaller I-ICC but substantially decreased standard deviations, depending on the assumed global variance and the degrees of freedom. With *τ* = 5 assumed for the 4 Holstein farms, the I-ICC estimates dropped by ∼14.4% to 23.7% for FP and 1.45% to 4.51% for PP, yet their standard deviations dropped very drastically by 80.0% to 91.7% for FP and 67.7% to 73.9%. The estimated outlier rates also dropped, yet remained in comparable ranges, when the data error rate was low (∼0.54%–0.04% for FP and 1.04%–0.14% for PP based on the 90th and 99.9th percentiles for the 4 Holstein farms). In general, using stable individual within-group variance allowed more conservative flagging of outliers with small to moderate error rates. Meanwhile, it can lead to a higher false negative rate, failing to detect true data anomalies, when using a substantially larger prior individual within-group variance than the global estimate and a large prior value of the degrees of freedom (e.g., *τ* > 10). With properly assumed prior values, the conclusions tend to be consistent using either method.

Second, a low I-ICC value does not always imply measurement or recording errors. Because $s_{i}^{2}$ captures all within-day variation, it can also increase under legitimate biological or management-related fluctuations, such as irregular milking intervals, incomplete milk let-down, or transient physiological changes. Consequently, the I-ICC should be interpreted as a consistency measure rather than a strict binary false versus positive test. Empirical threshold calibration, which helps distinguish true anomalies from expected variability, warrants further exploration.
